# A retrospective study on the efficacy and safety of Envafolimab, a PD-L1 inhibitor, in the treatment of advanced malignant solid tumors

**DOI:** 10.3389/fphar.2024.1356013

**Published:** 2024-01-31

**Authors:** Congjun Zhang, Jingjing Li, Hongyang Wu, Wei Huang, Liangshan Da, Yuanyuan Shen, Guoping Sun

**Affiliations:** Department of Medical Oncology, The First Affiliated Hospital of Anhui Medical University, Hefei, Anhui, China

**Keywords:** Envafolimab, PD-L1 inhibitor, advanced solid tumors, cancer treatment, immunotherapy

## Abstract

Envafolimab, a PD-L1 inhibitor, has demonstrated potential in treating advanced malignant solid tumors (AMST). To study its’ efficacy and safety in AMST, our retrospective study recruited 64 patients with various AMST, and treated with Envafolimab (400 mg every 3 weeks). We divided the patients into two cohorts: Cohort 1 (25 patients) receiving Envafolimab as first-line therapy, and Cohort 2 (39 patients) receiving it as second-line or subsequent therapy. Our analysis focused on Envafolimab’s efficacy and safety. Over a median follow-up of 7.1 months, Cohort I reported a Disease Control Rate (DCR) of 72.0% and an Objective response rate (ORR) of 12.0%, while Cohort II had a DCR of 51.3% and an ORR of 5.1%. Notably, patients with more than four treatment cycles showed higher DCR and longer Progression-Free Survival (PFS) than those with fewer cycles. Adverse events were observed in 68.8% of patients, with severe events (CTCAE grade 3/4) in 14.1%. Most adverse events were mild, leading to treatment discontinuation in only 3.1% of patients, with no life-threatening events reported. In summary, Envafolimab is a safe and effective treatment for AMST, in both initial and later therapy stages, particularly with extended treatment duration, meriting further clinical trials.

## 1 Introduction

Globally, there has been a notable increase in the prevalence of malignant tumors, and China has been a significant part of this trend. China has seen an alarming increase in both incidence and death rates linked to these malignant tumors. This positions it as one of the primary health issues confronting the nation. According to data from the 2019 National Death Cause Survey Report, malignant tumors have emerged as the leading cause of death, accounting for approximately 24.09% of all fatalities. Notably, cancers affecting vital organs like the lungs, liver, and stomach have played a major part in driving this statistic. Additionally, colorectal and esophageal cancers also contributed significantly to this distressing trend by constituting around 69.25%of reported causes (Feng et al., 2019; Han et al., 2022; Pan et al., 2023). Traditional therapeutic approaches including surgical procedures coupled with radiotherapy or chemotherapy are capable enough to target cancer cells but also inadvertently harm healthy tissues and cells, causing significant collateral damage that negatively affects patients’ quality of life leading to less than ideal treatment outcomes. Henceforth, there exists an immediate requirement for innovative treatments aimed at enhancing efficacy while ensuring safety, thereby improving long-term survival prospects particularly among those diagnosed with advanced solid tumor malignancies (Han et al., 2022).

Recent advancements made within immunotherapy-related research have led to substantial improvements regarding prognosis alongside elongating survival durations among cancer-affected individuals. The application of either singly or adjuvantly used immunotherapeutic agents has considerably decelerated disease progression while simultaneously uplifting overall patient wellbeing, thus emerging as a novel paradigm shift following surgery, radiation therapy, and chemo-based interventions (Mehdizadeh et al., 2021; Wan, Ryan, and Seymour, 2021). Immune Checkpoint Inhibitors (ICIs), a new generation of anti-cancer drugs, liberate the immune system enabling it to destroy tumorous growths by inhibiting negative costimulatory molecules like cytotoxic T lymphocyte-associated protein-4 (CTLA-4) and programmed death receptors (PD-1/PD-L1). Currently, numerous clinical trials indicate curative potential exhibited by immunotherapy. Following approval of Ipilimumab (a CTLA-4 inhibitor) by FDA for advanced melanoma, Nivolumab and Pembrolizumab (PD-1 inhibitors) for non-small cell lung cancer, renal carcinoma, and metastatic melanoma, immunotherapy has been recognized as the third systemic treatment option besides cytotoxic chemotherapy and targeted therapy (Sharma et al., 2017; Syn et al., 2017). However, intravenous administration of pembrolizumab, nivolumab, along with other PD-1/PD-L1 monoclonal antibodies can trigger severe immune-related adverse events (imAEs), potentially fatal infusion reactions thereby making the infusions themselves problematic (Kwok et al., 2016; Yau et al., 2023). This necessitates more effective, safe, easily administrable therapeutic drugs or regimens.

Envafolimab, a revolutionary fusion protein comprising two primary constituents: humanized single-domain anti-PD-L1 antibody combined with human immunoglobulin IgG1 Fc fragment, is the first recombinant humanized PD-L1 antibody to reach the clinical stage. It also holds the distinction of being the sole approved subcutaneous injectable immunotherapeutic agent, providing convenience for both patients and healthcare settings. Envafolimab exhibits excellent tissue penetration capabilities, ensuring uniform infiltration within tumorous tissues compared to regular monoclonal antibodies (Zhang et al., 2017). Pre-clinical studies reveal high concentrations of Envafolimab effectively inducing cytokine secretion in T-cells while demonstrating superior anti-tumor efficacy at comparable dosages against Durvalumab (Wang et al., 2023). Owing to its potential therapeutic benefits, the National Medical Products Administration (NMPA) granted approval to Envafolimab on 25 November 2020, for treating adult patients diagnosed with unresectable/metastatic solid tumors exhibiting high microsatellite instability (MSI-H)/deficient mismatch repair (dMMR).

Phase I clinical trial showed that the safety and pharmacokinetics of Envafolimab were similar to those of other traditional antibodies. In this trial, 28 patients with advanced solid tumors received subcutaneous Envafolimab (0.01–10 mg/kg) once a week during the dose-escalating phase (n = 18). The pharmacokinetic findings showed that there was no dose-limiting toxicity and that the median time to maximum Envafolimab plasma concentration was 4–7 days. Patients in the dose-finding phase (n = 10) were administered 300 mg of envafolimab subcutaneously every 4 weeks. In a stable state, the medication’s half-life was increased to 23 days. Hypothyroidism (14%), diarrhea (14%), nausea (18%), and fatigue (29%) were reported as the most frequent TEAEs. The three patients who experienced grade 3 adverse reactions did not exhibit any injection site reactions or grade IV drug-related adverse reactions (Papadopoulos et al., 2021) The safety, tolerance, and pharmacokinetics of subcutaneously injected Envafolimab was evaluated in a Japanese cohort of patients with advanced solid tumors. Envafolimab was well tolerated and importantly showed long-lasting anti-tumor activity in different regimens (Shimizu et al., 2022). A Phase II clinical study of Envafolimab combined with FOLFOX, as first-line treatment for advanced adenocarcinoma of stomach/esophagogastric junction, also showed good safety and tolerability, and clear preliminary anti-tumor effect (Xiang et al., 2012). Based on the existing cumulative data in the clinical setting, subcutaneous administration of Envafolimab is safe and tolerable in a wide dose range among subjects with advanced cancers (0.01 to 10 mg/kg Q1W). The versatility in dosing reflects the adaptability of Envafolimab, providing clinicians with a range of therapeutic options to tailor treatment according to patient needs and responses. No obvious dose independence was observed.

Thus, this retrospective study aims at evaluating the safety alongside effectiveness of Envafolimab when used in the management of advanced malignant solid tumors, thus offering valuable clinical insights benefiting a larger patient population affected by such malignancies.

## 2 Materials and methods

### 2.1 Dataset overview

The research conducted a retrospective examination of clinical data from patients suffering from advanced malignant solid tumors who were administered Envafolimab at the hospital affiliated to Department of Medical Oncology, spanning December 2021 to February 2023. The final follow-up was concluded on 16 February 2023. Prior to receiving Envafolimab treatment, all patients underwent extensive pre-treatment assessments including hematology tests, biochemistry analysis and other pertinent diagnostic examinations. Eligibility criteria included: 1) Age ≥18 years; 2) Cytologically or histopathologically confirmed advanced malignant solid tumors in stages III/IV; 3) At least one quantifiable lesion as per RECIST 1.1 guidelines; 4) An ECOG Performance Status score ranging between zero and one; and 5) Normal operation of major organs along with bone marrow healthiness. Exclusion parameters encompassed: previous experience of PD-L1/PD-1 inhibitor therapy, serious cardiopulmonary conditions or autoimmune diseases or immunodeficiency disorders alongside organ transplantation history; incomplete clinical records that hinder evaluation efficacy assessment plus adverse reaction occurrence frequency determination; and absence of follow-up information due to patient attrition. The study obtained approval from our hospital’s institutional ethics committee (Ethics Approval No.: AMU-FAH-EC-Fast-PJ2023-04-52), adhering strictly to the Declaration of Helsinki principles besides local legislation enforcement. Written informed consent was secured prior initiating any treatment procedure.

### 2.2 Therapeutic approaches

Patients received monotherapy with Envafolimab at different lines of therapy, which continued until disease progression detection, death eventuality, toxicity development manifestation, or withdrawal consent given by patient. Cohort 1 (25 patients) received Envafolimab as first-line therapy, and Cohort 2 (39 patients) received it as second-line or subsequent therapy. Envafolimab dosage regimen comprised subcutaneous administration every 3 weeks (400 mg/Q3W). This protocol deviated slightly from standard weekly doses but demonstrated safety measures coupled with effectiveness for treating unresectable MSI-H/dMMR colon cancer patients, as outlined by Wang et al. (2023)

### 2.3 Efficacy appraisal and adverse reactions

Effectiveness was gauged bi-monthly (every 2 cycles or a cycle equivalent to 3 weeks) using RECIST v1.1 guidelines. Assessment results were categorized into Complete Response (CR) - total clearance of all target lesions; Partial Response (PR) - reduction in sum diameters of baseline lesions by ≥ 30%; Progressive Disease (PD) - increment in the sum of diameters for baseline lesions by ≥ 20% or new lesion emergence; Stable Disease (SD) - neither sufficient shrinkage to qualify for PR nor adequate increase warranting PD categorization. Primary study outcomes included Objective Response Rate (ORR), Disease Control Rate (DCR), Progression-Free Survival (PFS), Overall Survival (OS). ORR is represented as the proportion of patients achieving CR/PR, while DCR includes patient count with CR, PR, and SD classification. PFS measures the time span from Envafolimab initiation until disease progression detection or death eventuality. OS calculates the duration between treatment commencement till death occurrence. Adverse reactions, including Treatment-Emergent Adverse Events (TEAEs) and Serious Adverse Events (SAEs), were recorded and classified according to NCI Common Terminology Criteria for Adverse Events (CTCAE) version 5. SAE category encompassed any TEAE resulting in fatality (excluding primary disease progression), life-threatening conditions manifestation, or extended hospital stay.

### 2.4 Statistical analysis

We used SPSS software (version 25, IBM United States) for performing the statistical analysis. Following examining normality test, normally distributed data was expressed as mean ± standard deviation (mean ± SD) and inter-group comparisons made utilizing t-tests. Non-normally distributed data were represented by the median (M) and interquartile range (IQR), with values at the 25th and 75th percentiles (P25, P75), and analyzed using the rank-sum test. Count data expression used number (%) representation followed by Chi-square testing/Fisher’s exact tests. Descriptive statistics applied to safety-related data. ORR and DCR were analyzed as binary variables (yes/no). Median follow-up time, PFS, and OS were estimated using Kaplan-Meier curves, with 95% confidence intervals for ORR, DCR calculated via the Clopper-Pearson method. DCR subjected to logistic regression analysis in a multivariate context while the Cox Proportional Hazards model applied to PFS data during multivariate analysis identifying independent prognostic factors. Statistical significance was set at a threshold of *p* ≤ 0.05.

## 3 Results

### 3.1 Baseline characteristics

This study involved conducting an analysis on the clinical data of a cohort comprising 64 patients who had received a diagnosis of advanced malignant solid tumors at stages III and IV. As shown in Table 1, 82.8% of the total population were male (n = 32: 23 cases in cohort 1 and 30 cases in cohort 2), while 17.2% were female (n = 11: 2 cases in cohort 1 and 9 cases in cohort 2). The age distribution exhibited considerable variability, ranging from 29 to 86 years (mean = 64.3 years). There was no statistically significant difference in the mean age between two groups. The individuals in this study were diagnosed with different types of cancer including lung cancer (cohort 1 = 5 cases and cohort 2 = 13 cases), gastric cancer (cohort 1 = 7 cases and cohort 2 = 5 cases), liver cancer (cohort 1 = 7 cases and cohort 2 = 4 cases), and esophageal cancer (cohort 1 = 2 cases and cohort 2 = 6 cases). Envafolimab was administered as a single-agent therapy in only three patients (4.7%; cohort 1 = 1 case and cohort 2 = 2 cases), which indicates its limited utilization within specific clinical contexts. A larger portion of the group (56.3%; cohort 1 = 19 cases and cohort 2 = 17 cases) received Envafolimab alongside chemotherapy agents like albumin - bound paclitaxel and oxaliplatin-showcasing a preference for integrated treatment approaches. Among these patients receiving targeted therapies were those treated with EGFR inhibitors like gefitinib and osimertinib - accounting for eighteen cases (28.1%; cohort 1 = 3 cases and cohort 2 = 15 cases). This highlights a growing trend towards precision medicine based on tumor genetics. Additionally noteworthy is that seven patients (10.9%; cohort 1 = 2 cases and cohort 2 = 5 cases) underwent combination treatments involving Envafolimab along with both chemotherapy and targeted therapy - indicating the implementation of multi-pronged strategies against more resistant tumor profiles. The duration of therapy exhibited variability across participants. Specifically, 40 cases (62.5%; cohort 1 = 14 cases and cohort 2 = 26 cases) underwent one to four cycles of treatment, while 24 cases (37.5%; cohort 1 = 11 cases and cohort 2 = 13 cases) underwent more than four cycles. This could possibly indicate either sustained positive responses or ongoing therapeutic plans (Table 1).

**TABLE 1:** Baseline characteristics of patients.

Baseline characteristics	Cohort 1	Cohort 2	n (%)
Age (years)	65.8 ± 10.4	63.3 ± 10.4	64.3 ± 10.4
Gender			
Male	23 (92.0)	30 (76.9)	53 (82.8)
Female	2 (8.0)	9 (23.1)	11 (17.2)
ECOG-PS			
0	3 (12.0)	14 (35.9)	17 (26.6)
1	22 (88.0)	25 (64.1)	47 (73.4)
Tumor type			
Lung cancer	5 (20.0)	13 (33.3)	18 (28.1)
Gastric cancer	7 (28.0)	5 (12.8)	12 (18.8)
Liver cancer/intrahepatic	7 (28.0)	4 (10.3)	11 (17.2)
Cholangiocarcinoma	2 (8.0)	6 (15.4)	8 (12.5)
Esophageal cancer	4 (16.0)	11 (28.2)	15 (23.4)
Other^a^			
Clinical staging			
III	6 (24.0)	7 (17.9)	13 (20.3)
IV	19 (76.0)	32 (82.1)	51 (79.7)
Treatment cycle			
1 to 4 cycles	14 (56.0)	26 (66.7)	40 (62.5)
5 and more cycles	11 (44.0)	13 (33.3)	24 (37.7)
Treatment regimens			
Envafolimab	1 (4.0)	2 (5.1)	3 (4.7)
Envafolimab plus chemotherapy	19 (76.0)	17 (43.6)	36 (56.3)
Envafolimab plus targeted therapy	3 (12.0)	15 (38.5)	18 (28.1)
Envafolimab plus chemotherapy and targeted therapy	2 (8.0)	5 (12.8)	7 (10.9)

^a^ Other encompasses various other malignancies such as intestinal, gallbladder, and urothelial cancers, as well as cancers of the parotid gland, anal canal, oral cavity, and cervix.

### 3.2 Comparative efficacy of envafolimab in first and second-line therapies

The effectiveness of envafolimab was evaluated in 64 patients as of 16 February 2023, with a median follow-up period of 7.1 months (ranging from 5.0 to 9.2 months). Envafolimab was used as a second-line or later therapy by most of the study’s subjects (n = 39,60.9%), highlighting its usefulness in treating situations where patients are resistant to previous treatments (Table 2). The efficacy evaluation, as shown in Table 2, revealed that of the patients on first-line therapy, 3 had a Partial Response (PR), 15 had Stable Disease (SD), and 7 had Progressive Disease (PD). This led to an Objective response rate (ORR) of 12.0% and a Disease Control Rate (DCR) of 72.0%. By comparison, patients receiving second-line therapy or higher did not show any CR; instead, 2 showed PR, 18 had SD, and 19 had PD, resulting in an ORR of 5.1% and a DCR of 51.3%. These results shed light on the disparate outcomes in this patient cohort according to the therapeutic line.

**TABLE 2:** Efficacy evaluation of patients who had advanced malignant solid tumor and received different therapy lines.

Number of therapy lines	Number of cases	CR	PR	SD	PD	ORR (%)	DCR (%)
First line	25	0	3	15	7	12.0	72.0
Second line and above	39	0	2	18	19	5.1	51.3

### 3.3 Impact of clinical stage, therapy line, and treatment cycles on response rates in envafolimab treated patients: Univariate analysis

The analysis of the ORR and DCR in a clinical study of Envafolimab for advanced malignant solid tumors revealed diverse outcomes depending on clinical staging, number of therapy lines, and treatment cycles. These findings are presented in Table 3 for ORR and Table 4 for DCR.

**TABLE 3:** Antitumor activities based on clinical staging, number of therapy lines, and treatment cycles.

Baseline clinical data	Number of cases	CR	PR	SD	PD	ORR/%	*χ^2*	*p*
Clinical staging [n (%)]								
III	13	0	2	7	4	2 (15.4)	0.314	0.575
IV	51	0	3	26	22	3 (5.9)		
Number of therapy lines [n (%)]								
First line	25	0	3	15	7	3 (12.0)	0.273	0.602
Second line and above	39	0	2	18	19	2 (5.1)		
Treatment cycle [n (%)]								
1 to 4 cycles	40	0	1	19	20	1 (2.5)	2.444	0.118
4 and more cycles	24	0	4	14	6	4 (16.7)		

**TABLE 4:** Univariate analysis and multivariate logistic regression analysis of DCR.

Baseline clinical data	Number of cases	DCR/%	*Univariate analysis*		*Multivariate analysis*	
			*X^2*	*p*	*OR (95%CI)*	*p*
**Clinical staging [n (%)]**			0.657	0.418		
** **III	13	9 (69.2)				
** **IV	51	29 (56.9)				
**Number of therapy lines [n (%)]**			2.711	0.100		
** **First line	25	18 (72.0)				
** **Second line and above	39	20 (51.3)				
**Treatment cycle [n (%)]**			3.887	0.049	3.632 (1.025-12.876)	0.046
** **1 to 4 cycles	40	20 (50.0)				
** **4 and more cycles	24	18 (75.0)				

The results of clinical staging analysis indicated that there was no statistically significant disparity in ORR between stage III and stage IV patients. Additionally, there was no significant difference observed in DCR between these two stages. No statistically significant differences were observed in ORR or in the univariate DCR analysis when comparing the number of therapy lines. However, the multivariate analysis revealed a significant difference in the DCR when comparing first-line treatment to second-line and subsequent treatments. In relation to the treatment cycles, individuals who underwent 1 to 4 cycles exhibited an ORR of 2.5% and a DCR of 50.0%. Conversely, patients who received more than 4 cycles demonstrated an ORR of 16.7% and a DCR of 75.0%. The observed discrepancy in DCR exhibited statistical significance, implying a potential correlation between an increased number of treatment cycles and improved disease control outcomes.

### 3.4 Impact of clinical stage, therapy line, and treatment cycles on response rates in envafolimab treated patients: Multivariate analyses

The treatment efficacy of Envafolimab in our cohort was statistically analyzed to determine the influence of clinical stage, treatment cycle, and number of therapy lines on PFS. As illustrated in Table 5, undergoing more than four treatment cycles significantly impacts PFS compared to patients who completed one to four cycles. The Hazard Ratio (HR) for patients receiving more than four cycles was 0.259, indicating a lower risk of progression or death for patients receiving an extended treatment. Furthermore, stage III and IV patients had median PFS of 3.7 and 3.5 months, respectively. The difference was not statistically significant. Patients who recieved Envafolimab as a first-line treatment had a median PFS of 5.4 months, compared to 3.2 months for those treated second-line or later.

**TABLE 5:** Univariate analysis and multivariate cox regression analysis of PFS.

Factor	Median PFS (month)		95%*CI*	*Univariate analysis*		*Multivariate analysis*	
				*X^2*	*p*	*HR (95%CI)*	*p*
**Clinical staging [n (%)]**				0.990	0.320		
** **III	3.7		0.0–7.6				
** **IV	3.5		0.9–6.0				
**Number of therapy lines [n (%)]**				4.240	0.039	1.943 (0.825-4.574)	0.129
** **First-line	5.4		-				
** **Second-line and above	3.2		2.3–4.0				
**Treatment cycle [n (%)]**				12.348	<0.001	0.259 (0.109-0.614)	0.002
** **1 to 4 cycles	2.6		2.1–3.1				
** **4 and more cycles	6.5		3.5–9.4				

This comprehensive analysis underscores the beneficial effects of a higher number of treatment cycles in patients receiving Envafolimab, both in terms of sustaining disease control and prolonging the time before disease progression.

### 3.5 Analysis of progression-free survival (PFS) based on clinical stages and treatment lines

**FIGURE 1:**
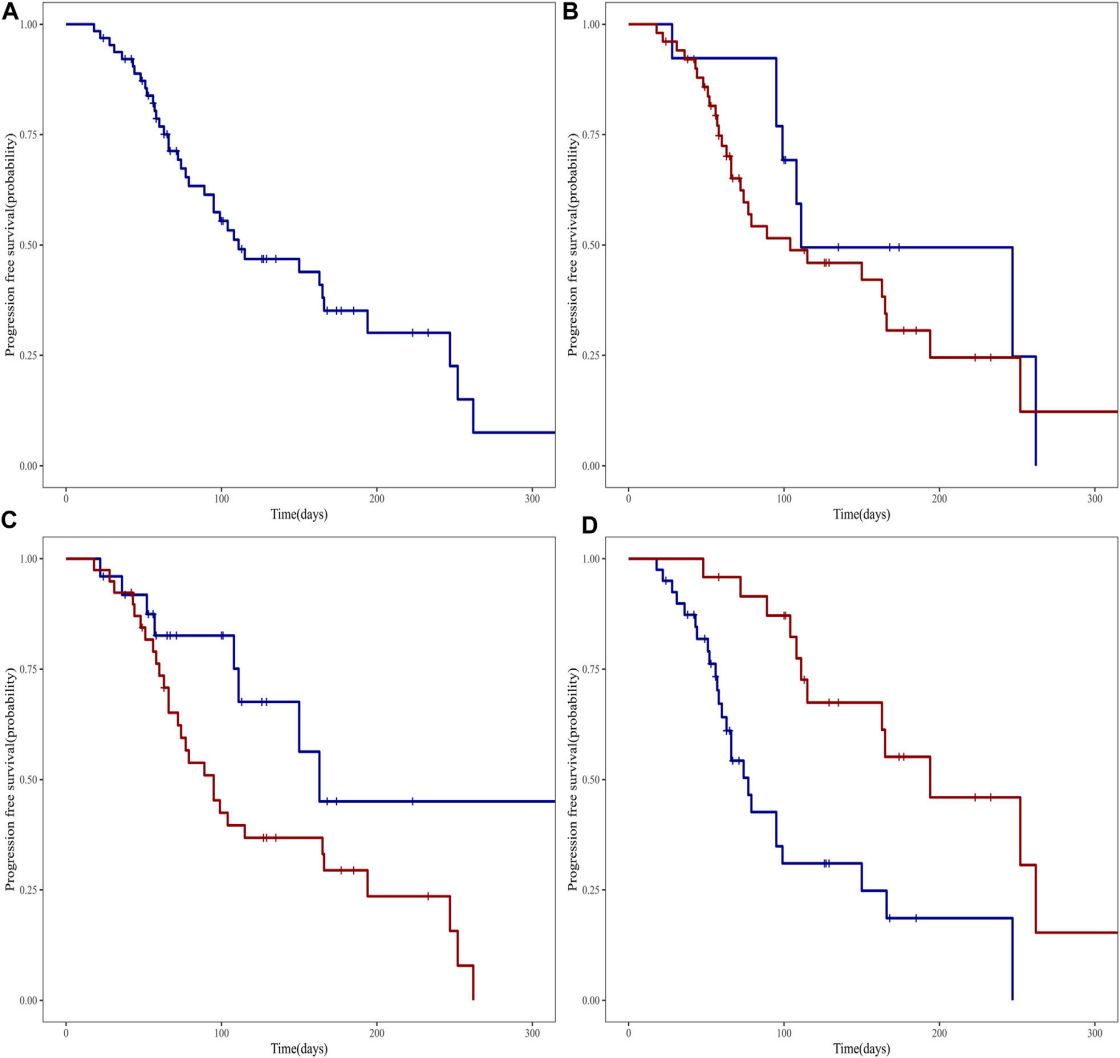
PFS survival curve of patients with advanced malignant solid tumors treated with Envafolimab: **(A)** PFS survival curve of all patients; **(B)** PFS survival curves of patients with different clinical stages (blue line represents stage III and red line represents stage IV); **(C)** PFS survival curves of patients receiving different lines of therapy (blue line represents the first line, red line represents the second line and above); **(D)** PFS survival curve of patients receiving different treatment cycles (blue line represents patients receiving 1 to 4 cycles, red line represents patients receiving more than 4 cycles).

Figure 1A illustrates the study of median PFS in 64 patients treated with Envafolimab. The entire cohort’s median PFS was 3.7 months. Patients with stage III cancer had a median PFS of 3.7 months, while those with stage IV cancer experienced a median PFS of 3.5 months. However, statistical analysis showed no significant difference between these groups (*p* = 0.320), as depicted in Figure 1B.

The effect of treatment lines on median PFS was also analyzed. Patients receiving first-line therapy had a median PFS of 5.4 months, longer than the approximate 3.2 months in patients undergoing second-line or subsequent therapies, as indicated in Figure 1C. Furthermore, number of treatment cycles also affected PFS outcomes. Those who received 1 to 4 cycles had a median PFS of approximately 2.6 months. In contrast, patients treated for more than 4 cycles showed a longer median PFS of about 6.5 months (*p* < 0.001), as shown in Figure 1D. The median OS remained undetermined, suggesting the need for extended observation to assess potential long-term survival benefits.

### 3.6 Adverse events in patients treated with envafolimab for advanced malignant solid tumors

In this study, 95.3% of patients (61 out of 64) experienced varying TEAEs, detailed in Table 6. Anemia, decreased platelet count, Loss of appetite, increased transaminases, hypokalemia, decreased glomerular filtration rate, and increased blood bilirubin detected as most common adverse events with 30% or higher incidence. 29.7% of patients (19 out of 64) had severe TEAEs of CTCAE grade 3 to 4, with decreased platelet count and white blood cell count being the most common with incidence of 5% or higher. Note that the study had no grade 5 TEAEs. 20.3% of patients (13 out of 64) showed serious adverse events (SAEs) like enteritis, pulmonary infection, obstructive jaundice, upper gastrointestinal bleeding, myelosuppression, rash, hypothyroidism, infective endocarditis, and nephritis. 68.8% of these events (44 out of 64) were TEAEs related to the administration of Envafolimab. For TEAEs specifically related to Envafolimab, the incidence of CTCAE grade 3/4 events was 14.1% (9 out of 64), with no individual type of TEAE surpassing the 5% incidence mark. Events that occurred at an incidence of 1% or more included anemia, increased blood bilirubin, increased transaminases, decreased platelet count, decreased white blood cell count, loss of appetite, fever, rash, and pulmonary infection. Due to adverse reactions related to Envafolimab, 3.1% of patients (2 out of 64) discontinued the treatment. One patient ceased treatment due to immune-related liver injury, and another due to a severe rash on the back. However, no life-threatening TEAEs were reported.

**TABLE 6:** The incidence of all grades of TEAEs related to Envafolimab ≥10%.

Preferred term	Any severity	Maximum severity	Preferred term
		CTCAE grade 3/4	CTCAE grade 5
TEAEs related to Envafolimab	44 (68.8)	9 (14.1)	0
Hematological toxicity			
Anemia	17 (26.6)	2 (3.1)	0
Platelet count decreased	14 (21.9)	1 (1.6)	0
White blood cell count decreased	11 (17.2)	1 (1.6)	0
Non-hematological toxicity			
Loss of appetite	18 (28.1)	1 (1.6)	0
Blood bilirubin increased	16 (25.0)	2 (3.1)	0
Transaminases increased	15 (23.4)	2 (3.1)	0
Hyperglycemia	15 (23.4)	0	0
Asthenia	12 (18.8)	0	0
Glomerular filtration rate decreased	11 (17.2)	0	0
Nausea	9 (14.1)	0	0
Fever	9 (14.1)	1 (1.6)	0
Skin rash	7 (10.9)	1 (1.6)	0
Creatinine increased	7 (10.9)	0	0

Injection site reactions were observed in 3.1% of patients (2 out of 64), presenting as skin redness or rash with itching, but all were classified as mild to moderate in severity (grade 1/2), as detailed in Table 6. This safety profile emphasizes the need for careful monitoring of patients receiving Envafolimab, particularly for hematological toxicity and liver function disturbances.

In our study, the management of TEAEs due to Envafolimab, a common issue in cancer treatment with PD-L1 inhibitors, involved temporarily interrupting the drug administration until the TEAE improved to Grade 0-1. For severe cases (CTCAE grade 3/4), we might consider permanently discontinuing the treatment. Additionally, corticosteroids were used for managing immune-related adverse events. These approaches align with standard oncological practices for ensuring patient safety and effective treatment management.

## 4 Discussion

In our research, we assessed the effectiveness and safety of Envafolimab in addressing advanced malignant solid tumors. The results indicated that both standalone treatment and combined therapy with Envafolimab displayed a manageable safety record. The incidence and severity of TEAEs were basically consistent with the known adverse reactions of chemotherapy, targeted therapy, and Envafolimab, and no new safety concerns were observed. A total of 68.8% of patients had TEAEs related to Envafolimab. Although the incidence of grade-3/4 TEAEs related to Envafolimab reached 14.1%, the incidence of all adverse reactions was lower than 5%, and no grade-5 TEAEs related to Envafolimab occurred. Two patients discontinued Envafolimab due to adverse reactions related to Envafolimab, and their symptoms improved after stopping treatment.

Clinically, Envafolimab, particularly as a second-line therapy, showed promising outcomes. The ORR, DCR, and PFS were favorable, suggesting that Envafolimab could offer durable responses, essential for patients with advanced diseases. This aligns with similar studies, reinforcing PD-L1 inhibition’s role in treating MSI-H/dMMR advanced solid tumors (Li et al., 2021). With a median follow-up period of 11.5 months, this study included a total of 103 patients diagnosed with advanced cancer. The ORR for patients with advanced solid tumors, advanced gastric cancer (GC), advanced colorectal cancer (CRC), and other solid tumors were reported as 44.7%, 55.6%, 40.0%, and 43.1%, respectively. The median DOR was not achieved, and the 12-month DOR rates of patients with advanced CRC, advanced GC, and other solid tumors and all populations were 88.4%, 100%, 100%, and 92.2% respectively. The median PFS was 11.1 months. The median OS was not achieved, and the 12-month OS rates of patients with advanced CRC, advanced GC, and other solid tumors and all populations were 72.9%, 83.3%, 75.0%, and 74.6% respectively. These results are particularly compelling, suggesting that Envafolimab may offer durable responses in a clinical setting, a crucial factor for patients with advanced disease. The incidence of all grades of TEAEs was 96%, with 16 patients (15.5%) having Grades 3/4 drug related TEAEs. Although no study drug-related grade-5 TEAEs were observed, 2.9% of the subjects stopped treatment permanently due to drug-related TEAEs. The incidence of injection site reaction was low (8.7%) graded-1/2, and there were no related SAEs or events leading to permanent drug withdrawal (Li et al., 2021).

In our study, Envafolimab also showed a clear anti-tumor effect in the clinical setting. The ORR, DCR, and median PFS of patients with advanced malignant solid tumors that received Envafolimab as the first-line therapy were 12.0%, 72.0%, and 5.4 months respectively. A notable aspect of our study is the efficacy of Envafolimab in a broader patient population, including various tumor types beyond the MSI-H/dMMR subset. Despite the lower efficacy rates compared to the aforementioned Phase II study, the outcomes underscore the potential of Envafolimab to provide clinical benefit across various tumor types and treatment lines. After failing curative treatment intent for advanced malignant solid tumors with the first-line systemic standard of care, patients received Envafolimab and had a resulting ORR, DCR, and median PFS of 5.1%, 51.3% and 3.2 months respectively, showing that Envafolimab had good outcome both as the first-line therapy and the back-line therapy for extensive cancer types. The observed disparity in efficacy rates may be partly explained by the broader and more heterogeneous patient population in this study, which included tumor types beyond the MSI-H/dMMR subset known to be particularly responsive to PD-1/PD-L1 blockade (Le et al., 2015; Bonneville et al., 2017). The two cancers that had the highest incidence (>10%) were GC and CRC (Amonkar et al., 2019). Clinical outcomes, however, might not be as successful as those documented in clinical trials since most patients in real-world practice might not be able to undergo mismatch repair (MMR) or microsatellite instability (MSI) detection. Moreover, this study showed that there was no statistical difference in ORR, DCR, and PFS between patients with different clinical stages, and the DCR and PFS of patients treated for more than 4 cycles were higher than those treated for 4 cycles or less indicating that Envafolimab can be advantageous in treating malignant solid tumors, with longer the treatment duration providing the higher curative effect and better prognosis.

Furthermore, Envafolimab’s TEAEs showed a similar incidence rate to other PD-1 inhibitors in this category, with about 20% of patients experiencing grade ≥3 TEAEs and 10% facing immune-related reactions. Unlike pembrolizumab, which caused immune enteritis and pneumonia in 2%–7% and 1%–5% of patients respectively, these conditions were not observed in those treated with Envafolimab. This distinction could be linked to the different interactions between PD-1 and PD-L2. Considering serious immune-related TEAEs as a limiting factor in immunotherapy, Envafolimab appears more suitable for high-risk groups like elderly patients with interstitial lung diseases (Xiao et al., 2014; Yu et al., 2019; Chen et al., 2022). Unlike pembrolizumab and other approved PD-1/PD-L1 antibodies administered intravenously-potentially leading to life-threatening infusion reactions-Envafolimab is given subcutaneously without such reactions (Kwok et al., 2016). In this study, 29.7% of patients switched to Envafolimab following the failure of other PD-1 inhibitors, mainly due to disease progression or immune-related TEAEs, suggesting its potential as a preferred option post-failure of other ICIs. Envafolimab’s subcutaneous administration, taking only 30 s, offers greater convenience and patient compliance, particularly for those needing long-term therapy. It can be administered by community doctors or at home, enhancing patients’ quality of life. Its 400 mg Q3W dosage further simplifies treatment compared to the standard 150 mg QW regimen. Additionally, Envafolimab’s high stability and non-reliance on cold-chain transportation reduce the medical resource burden. However, this single-center retrospective study’s biases necessitate further large-scale, multi-center randomized controlled trials, focusing on specific cancer types, treatment regimens, and therapy lines to optimize Envafolimab treatment strategies.

A comparison between the safety profiles of Envafolimab and other PD-L1 inhibitors has revealed interesting insights. Our research highlights a 68.8% occurrence rate of adverse events associated with Envafolimab, with 14.1% classified as severe under the CTCAE grade 3/4 criteria. In contrast, a study on Atezolizumab treatment in urothelial carcinoma patients reported a similar incidence rate of about 69%, with severity at levels 3/4 accounting for 15% (Rosenberg et al., 2016). Research into Pembrolizumab showed that approximately 60% of bladder cancer patient cohorts experienced adverse events, with 17% categorized as level 3 severity or higher (Bellmunt et al., 2017). Another comprehensive evaluation revealed that Nivolumab is linked to an adverse event rate of 71% among non-small cell lung cancer patients, albeit with only 10% being severe (Borghaei et al., 2015). These comparisons indicate that Envafolimab’s safety profile is comparable to that of existing PD-L1 inhibitors, potentially offering a slightly lower incidence of severe adverse events in some instances.

## 5 Conclusion

In conclusion, the data from this study and comparative analyses with current research affirm that Envafolimab is a safe and effective treatment option for advanced malignant solid tumors, providing a viable alternative to intravenous immunotherapy. For patients who need long-term care, in particular, the ease of its delivery by subcutaneous administration along with a patient-friendly dosage schedule may improve patient compliance and quality of life. Future research should continue to refine the indications and administration protocols for Envafolimab, ensuring that patients receive the most effective, personalized care possible.

## Data Availability

The original contributions presented in the study are included in the article/Supplementary Material, further inquiries can be directed to the corresponding author.

## References

[B1] Amonkar M. Lorenzi M. Zhang J. Mehta S. Liaw K.-L. (2019). 'Structured literature review (SLR) and meta-analyses of the prevalence of microsatellite instability high (MSI-H) and deficient mismatch repair (dMMR) in gastric, colorectal, and esophageal cancers. J. Clin. Oncol. 37, e15074–e74. 10.1200/jco.2019.37.15_suppl.e15074

[B2] Bellmunt J. de Wit R. Vaughn D. J. Fradet Y. Lee J.-L. Fong L. (2017). 'Pembrolizumab as second-line therapy for advanced urothelial carcinoma. N. Engl. J. Med. 376, 1015–1026. 10.1056/NEJMoa1613683 28212060 PMC5635424

[B3] Bonneville R. Krook M. A. Kautto E. A. Miya J. Wing M. R. Chen H. Z. (2017). Landscape of microsatellite instability across 39 cancer types. JCO Precis Oncol. 10.1200/PO.17.00073 PMC5972025 29850653

[B4] Borghaei H. Paz-Ares L. Horn L. Spigel D. R. Martin S. Ready N. E. (2015). 'Nivolumab versus docetaxel in advanced nonsquamous non–small-cell lung cancer. N. Engl. J. Med. 373, 1627–1639. 10.1056/NEJMoa1507643 26412456 PMC5705936

[B5] Chen M. Jiang M. Wang X. Shen L. Li J. (2022). 'Envafolimab - first PD-1/PD-L1 antibody to be administered by subcutaneous injection for microsatellite instability-high or deficient mismatch repair advanced solid tumors. Expert Opin. Biol. Ther. 22, 1227–1232. 10.1080/14712598.2022.2125799 36124972

[B6] Feng R.-M. Zong Y.-N. Cao S.-M. Xu R.-H. (2019). Current cancer situation in China: good or bad news from the 2018 Global Cancer Statistics? Cancer Commun. 39, 22. 10.1186/s40880-019-0368-6 PMC6487510 31030667

[B7] Han X. Wang Z. Huang D. Deng K. Wang Q. Li C. (2022). 'Analysis of the disease burden trend of malignant tumors of the female reproductive system in China from 2006 to 2020. BMC Women's Health 22, 504. 10.1186/s12905-022-02104-2 36476597 PMC9730658

[B8] Kwok G. Yau T. C. Chiu J. W. Tse E. Kwong Y. L. (2016). Pembrolizumab (keytruda). Hum. Vaccin Immunother. 12, 2777–2789. 10.1080/21645515.2016.1199310 27398650 PMC5137544

[B9] Le D. T. Uram J. N. Wang H. Bartlett B. R. Kemberling H. Eyring A. D. (2015). 'PD-1 blockade in tumors with mismatch-repair deficiency. N. Engl. J. Med. 372, 2509–2520. 10.1056/NEJMoa1500596 26028255 PMC4481136

[B10] Li J. Deng Y. Zhang W. Zhou A. P. Guo W. Yang J. (2021). 'Subcutaneous envafolimab monotherapy in patients with advanced defective mismatch repair/microsatellite instability high solid tumors. J. Hematol. Oncol. 14, 95. 10.1186/s13045-021-01095-1 34154614 PMC8218452

[B11] Mehdizadeh S. Bayatipoor H. Pashangzadeh S. Jafarpour R. Shojaei Z. Motallebnezhad M. (2021). 'Immune checkpoints and cancer development: therapeutic implications and future directions. Pathol. Res. Pract. 223, 153485. 10.1016/j.prp.2021.153485 34022684

[B12] Pan Z. Yu L. Shao M. Ma Y. Cheng Y. Wu Y. (2023). 'The influence of meteorological factors and total malignant tumor health risk in Wuhu city in the context of climate change. BMC Public Health 23, 346. 10.1186/s12889-023-15200-1 36797719 PMC9933274

[B13] Papadopoulos K. P. Harb W. Peer C. J. Hua Q. Xu S. Lu H. (2021). 'First-in-Human phase I study of envafolimab, a novel subcutaneous single-domain anti-PD-L1 antibody, in patients with advanced solid tumors. Oncologist 26, e1514–e1525. 10.1002/onco.13817 33973293 PMC8417852

[B14] Rosenberg J. E. Hoffman-Censits J. Powles T. van der Heijden M. S. Balar A. V. Necchi A. (2016). 'Atezolizumab in patients with locally advanced and metastatic urothelial carcinoma who have progressed following treatment with platinum-based chemotherapy: a single-arm, multicentre, phase 2 trial. Lancet 387, 1909–1920. 10.1016/S0140-6736(16)00561-4 26952546 PMC5480242

[B15] Sharma P. Hu-Lieskovan S. Wargo J. A. Ribas A. (2017). 'Primary, adaptive, and acquired resistance to cancer immunotherapy. Cell 168, 707–723. 10.1016/j.cell.2017.01.017 28187290 PMC5391692

[B16] Shimizu T. Nakajima T. E. Yamamoto N. Yonemori K. Koyama T. Kondo S. (2022). 'Phase I study of envafolimab (KN035), a novel subcutaneous single-domain anti-PD-L1 monoclonal antibody, in Japanese patients with advanced solid tumors. Invest. New Drugs 40, 1021–1031. 10.1007/s10637-022-01287-7 35932387

[B17] Syn N. L. Teng M. W. L. Mok T. S. K. Soo R. A. (2017). 'De-novo and acquired resistance to immune checkpoint targeting. Lancet Oncol. 18, e731–e741. 10.1016/S1470-2045(17)30607-1 29208439

[B18] Wan P. K. Ryan A. J. Seymour L. W. (2021). 'Beyond cancer cells: targeting the tumor microenvironment with gene therapy and armed oncolytic virus. Mol. Ther. 29, 1668–1682. 10.1016/j.ymthe.2021.04.015 33845199 PMC8116634

[B19] Wang L. Mou H. Hou X. Liao Q. (2023). 'Case report: a case of complete clinical response in a patient experiencing high microsatellite instability unresectable colon cancer being treated with a PD-L1 inhibitor after interstitial pneumonia. Front. Oncol. 13, 1126769. 10.3389/fonc.2023.1126769 36998453 PMC10043298

[B20] Xiang X. J. Liu Y. W. Zhang L. Qiu F. Yu F. Zhan Z. Y. (2012). 'A phase II study of modified FOLFOX as first-line chemotherapy in advanced small bowel adenocarcinoma. Anticancer Drugs 23, 561–566. 10.1097/CAD.0b013e328350dd0d 22481063

[B21] Xiao Y. Yu S. Zhu B. Bedoret D. Bu X. Francisco L. M. (2014). 'RGMb is a novel binding partner for PD-L2 and its engagement with PD-L2 promotes respiratory tolerance. J. Exp. Med. 211, 943–959. 10.1084/jem.20130790 24752301 PMC4010901

[B22] Yau T. Zagonel V. Santoro A. Acosta-Rivera M. Choo S. P. Matilla A. (2023). 'Nivolumab plus cabozantinib with or without Ipilimumab for advanced hepatocellular carcinoma: results from cohort 6 of the CheckMate 040 trial. J. Clin. Oncol. 41, 1747–1757. 10.1200/JCO.22.00972 36512738 PMC10022845

[B23] Yu S. Leung K. M. Kim H. Y. Umetsu S. E. Xiao Y. Albacker L. A. (2019). 'Blockade of RGMb inhibits allergen-induced airways disease. J. Allergy Clin. Immunol. 144, 94–108. 10.1016/j.jaci.2018.12.1022 30703386 PMC8088837

[B24] Zhang F. Wei H. Wang X. Bai Y. Wang P. Wu J. (2017). 'Structural basis of a novel PD-L1 nanobody for immune checkpoint blockade. Cell Discov. 3, 17004. 10.1038/celldisc.2017.4 28280600 PMC5341541

